# Emergency Department Visits and Inpatient Admissions Associated with Priapism among Males with Sickle Cell Disease in the United States, 2006–2010

**DOI:** 10.1371/journal.pone.0153257

**Published:** 2016-04-14

**Authors:** Brandi Dupervil, Scott Grosse, Arthur Burnett, Christopher Parker

**Affiliations:** 1 Division of Blood Disorders, National Center on Birth Defects and Developmental Disabilities, Centers for Disease Control and Prevention, Atlanta, GA, United States of America; 2 The James Buchanan Brady Urological Institute, Johns Hopkins Medical Institutions, Baltimore, MD, United States of America; National Cancer Institute, UNITED STATES

## Abstract

People with sickle cell disease (SCD) suffer from numerous acute complications that can result in multiple hospitalizations and emergency department (ED) and outpatient care visits. Priapism, a prolonged unwanted erection of the penis not due to sexual stimulation, is a serious complication among males with SCD. Variations in estimates of prevalence make it difficult to accurately assess the burden of this complication of SCD. We analyzed data from the Nationwide Emergency Department Sample (NEDS), a product of the Healthcare Cost and Utilization Project, for the years 2006 through 2010 to measure the numbers of ED visits and to examine patterns of subsequent hospitalizations associated with priapism among male patients with SCD. We find that among ED visits associated with males with SCD, those prompted by priapism are more likely to result in hospitalization than are those associated with pain.

## Introduction

Sickle cell disease (SCD) is an inherited disorder of hemoglobin that is estimated to affect 70,000 to 100,000 Americans [1, 2]. People with SCD suffer from numerous acute complications that may require acute care [3, 4]. One serious complication of SCD among males is priapism, which is a prolonged, unwanted, and often painful erection of the penis not due to sexual stimulation. An erection lasting more than 4 hours is classified as a major priapism episode and considered clinically hazardous[5,6]. Failure to treat a major episode of priapism can yield irreversible erectile complications, such as erectile dysfunction, disfigurement, or scarring, or a combination thereof [7–9].

Since 1934, priapism has been recognized as a complication of SCD among males[10]. Approximately 35–40% of males with homozygous SCD report having experienced priapism, with an age of onset typically between 15 and 20 years [8]. Many males with SCD experience stuttering priapism events, which are usually nocturnal, last 3–4 hours, are often relieved at home by simple physical measures (self-management), and allow normal erectile function between stuttering episodes. Priapism also has been associated with other severe complications of SCD, and it can result in subsequent physical, psychological, and emotional morbidities that further exacerbate the condition [5, 7, 11].

Over the past three decades, the characteristics of patients with SCD who experience priapism have been described [12, 13]. Psychological analysis has found that men with SCD who experience priapism often feel embarrassed and experience low self-esteem and decreased sexual desire [12]. Patients with SCD who have experienced priapism have lower hemoglobin F levels; higher levels of lactate dehydrogenase, bilirubin, and aspartate aminotransferase; and higher platelet counts [13].

Few health services research studies about priapism in the United States have been published [14–16]. Two studies reported that among ED visits associated with a diagnosis of priapism patients with SCD were roughly twice as likely to be admitted [14, 15]. A study of inpatient hospitalizations associated with a diagnosis of priapism reported that 42% were among those with SCD and their modal age of 18–24 years was much younger than for admissions with priapism among males without SCD [16].

The purpose of this paper is to compare characteristics of ED visits among male US residents with SCD who did or did not have a diagnosis of priapism reported in order to better understand how priapism compares with other acute and chronic complications of SCD in provoking acute hospital care. No previous study that we are aware of has compared priapism with other SCD complications as risk factors for inpatient admission from the ED. In particular, we hypothesized that males with SCD who present to the ED with priapism are more likely to be admitted than most other patients with SCD but less likely to be admitted than those with severe complications of SCD. This paper used a national sample of ED visits for the years 2006 through 2010 to estimate rates of ED visits and inpatient admission among males with SCD.

## Methods

### Data Source

Data from the Nationwide Emergency Department Sample (NEDS), a product of the Healthcare Cost and Utilization Project (HCUP) of the Agency for Healthcare Research and Quality (AHRQ), were abstracted to identify males with SCD who presented in an ED. NEDS, constructed using records from both the HCUP State Emergency Department Databases and the State Inpatient Databases, is the largest all-payer database in the United States [17]. All patient records/information in NEDS are de-identified. The HCUP databases are consistent with the definition of "limited data sets" under the HIPAA Privacy Rule and contain no direct patient identifiers. In accordance with federal legislation regarding the use of publically available population-based data, this study was exempt from institutional review board approval.

The database contains a total of 25 to 30 million (unweighted) records for ED visits at more than 950 hospitals. Each year, NEDS accounts for a stratified sample of approximately 20% of the hospital-based ED visits in the United States. SCD-related ED visits were stratified further by the presence or absence of an ICD-9-CM code of 607.3 for priapism.

### Population Sample

ED visits from 2006 through 2010 among males that included a discharge diagnosis of SCD were retrospectively abstracted using *International Classification of Diseases*, *9*^*th*^ *Revision*, *Clinical Modification* (*ICD-9-CM*) codes 282.6x, 282.41, or 282.42. A visit was considered SCD related whenever one of these ICD-9-CM was present. Each patient could have up to 15 diagnosis codes. Because of the complex sample design, **s**tratum weights were applied to discharges based on the year from which the discharge was drawn in order to obtain nationally representative estimates [17].

### ED Visit Characteristics

For this analysis, age, hospital region (Northeast, Midwest, South, West), month of admission, and day of the week of admission were used. Season of admission was calculated using the admission month variable, with the months December through February classified as winter, March through May as spring, June through August as summer, and September through November as autumn.

### Data Analysis

Statistical analyses were performed using SAS version 9.3. ED visits were grouped according to the presence or absence of concurrent priapism ICD-9-CM codes. Mean (M) and standard error (SE) values were calculated for continuous variables using the SURVEYMEANS Procedure, which adjusts for the NEDS survey design in developing variance estimates. Frequencies and percentages were calculated for categorical variables using the SURVEYFREQ procedure, which also adjusts for the NEDS survey design. Differences in the distributions and percentages of applicable characteristics were assessed using the Rao-Scott chi-square test [18]. The Rao-Scott chi-square test, a modified version of the Pearson’s chi-square test, accounts for complex sampling designs, such as that used in NEDS.

## Results

### Prevalence of ED Visits and Hospitalizations

The distributions of all ED visits and of ED visits leading to hospital admission for males with SCD are presented in Table 1. For the period 2006 through 2010, 584,652 weighted ED visits among males with SCD were estimated to have occurred in the United States. Of these, 231,692 (40%) visits resulted in patients being admitted to a hospital (either to the same hospital or a different hospital). Priapism occurred in only 2.3% (5,371/231,692) of ED visits resulting in admission, but the percentage of ED visits resulting in admission was higher for visits associated with priapism (50%) than visits without priapism (39%).

**Table 1:** Selected characteristics of emergency department visits and hospitalizations by patients with sickle cell disease, 2006–2010.

	Total ED Visits	Total ED Visits Admitted to the Hospital	Priapism related		Nonpriapism Related	
			*ED Visits*	*ED Visits Admitted to the hospital*	*ED Visits*	*ED Visits Admitted to the hospital*
**Number (row %)**	584,652	231,692 (39.6%)	10,788 (1.8%)	5,371 (49.8%)	573,863 (98.2%)	226,321 (39.4%)
**Mean Age (Standard Error)**	27 (0.04)	26 (0.07)	24 (0.18)	24 (0.27)^1^	27 (0.04)	26 (0.07)^1^
	***# (column %)***	***# (column %)***	***# (column %)***	***# (column %)***	***# (column %)***	***# (column %)***
**Age (years)**						
0–10	76,676 (13.1%)	33,502 (14.5%)	387 (3.6%)	267 (5%)	76,288 (13.3%)	33,235 (14.7%)
11–20	93,955 (16.1%)	43,330 (18.7%)	3,094 (28.7%)	1,682 (31.3%)	90,861 (15.8%)	41,648 (18.4%)
21–30	206,479 (35.3%)	75,890 (32.8%)	5,001 (46.4%)	2,349 (43.7%)	201,478 (35.1%)	73,541 (32.5%)
31–40	114,436 (19.6%)	41,300 (17.8%)	1,845 (17.1%)	856 (15.9%)	112,591 (19.6%)	40,444 (17.9%)
41–50	68,783 (11.8%)	26,010 (11.2%)	416 (3.9%)	188 (3.5%)	68,367 (11.9%)	25,822 (11.4%)
51–60	20,007 (3.4%)	8,704 (3.8%)	32 (0.3%)	24 (0.5%)	19,975 (3.5%)	8,680 (3.8%)
≥61	4,296 (0.7%)	2,936 (1.3%)	13 (0.1%)	5 (0.1%)	4,283 (0.8%)	2,931 (1.3%)
Not Stated	20	20			20	20
**Region**						
Northeast	113,311 (19.4%)	50,661 (21.9%)	2,318 (21.5%)	1,284 (23.9%)	110,993 (19.3%)	49,376 (21.8%)
Midwest	94,622 (16.2%)	38,516 (16.6%)	1,368 (12.7%)	677 (12.6%)	93,254 (16.3%)	37,839 (16.7%)
South	320,169 (54.8%)	121,179 (52.3%)	6,209 (57.6%)	3,055 (56.9%)	313,960 (54.7%)	118,124 (52.2%)
West	56,550 (9.7%)	21,336 (9.2%)	893 (8.28%)	355 (6.6%)	55,656 (9.7%)	20,982 (9.3%)
**Weekend**						
Yes	167,044 (28.6%)	64,299 (27.8%)	3,266 (30.3%)	1,555 (28.9%)	163,779 (28.6%)	62,744 (27.7%)
No	416,704 (71.4%)	167,378 (72.2%)	7,512 (69.7%)	3,816 (71.1%)	409,191 (71.4%)	163,561 (72.3%)
Not Stated	904	15	10		893	16
**Season**						
Winter	119,486 (24.8%)	45,275 (25.4%)	2,121 (23.2%)	1,141 (25.8%)^1^	117,068 (24.8%)	44,134 (25.4%)^1^
Spring	120,310 (25.0%)	44,614 (25.0%)	2,260 (24.8%)	1,176 (26.6%)^1^	118,050 (25.0%)	43,438 (25.0%)^1^
Summer	119,486 (24.9%)	43,408 (24.4%)	2,604 (28.5%)	1,171 (26.4%)^1^	116,882 (24.8%)	42,237 (24.3%)^1^
Fall	121,614 (25.3%)	44,929 (25.2%)	2,139 (23.4%)	942 (21.3%)^1^	119,475 (25.3%)	43,987 (25.3%)^1^
Not Stated	103,756	53,466	1,644	941	102,388	52,525

^1^ denotes a significant difference between hospital admissions for patients with priapism and without priapism at the < .05 level

Abbreviations: ED, emergency department; SCD, sickle cell disease; CI, confidence interval; SE, standard error. Figures for origin “Not stated” are included in All totals but are not distributed among specified origins or used to calculate percentages.

### Characteristics of SCD Inpatient Admissions With or Without Priapism

Among ED visits resulting in inpatient admission, visits with an associated priapism diagnosis occurred more among younger patients compared to SCD visits without priapism (p = 0.0001); the mean age of patients with SCD and priapism who were admitted to a hospital from an ED (M = 24 years, SE = 0.27) was 2 years younger than that of patients with SCD and no priapism who were admitted to a hospital from an ED (M = 26 years, SE = 0.07) (Table 1).

There were no significant differences in the regional distributions of admissions or the proportions of weekend/weekday admissions among those with priapism and those without (Table 1). However, a significant relationship was found between ED visits among patients with an associated diagnosis of priapism with the urbanicity of their county of residence with hospitalization compared to ED visits among patients with no associated priapism diagnosis (p ≤ .0004).

Additionally, a significant difference was detected between the season of admission and the presence or absence of priapism. The percentage of total hospitalizations from ED visits in a year associated with priapism was lowest in autumn (21%), but was higher—and nearly identical—for the other three seasons (27% in the spring and 26% in the winter and summer) (Table 1).

### Characteristics of Inpatient Admission Rates With or Without Priapism

The percentage of inpatient admissions from ED visits varied by region, although not significantly (Table 2). The highest percentage of admission for SCD ED visits with priapism was in the Northeast, 55%, and the lowest was in the West, 40%. For SCD ED visits without priapism, the highest percentage of hospital admission was also in the Northeast, 44%, and the lowest percentage was in the South and West, 38%.

**Table 2:** Admission rates from sickle cell disease-related emergency department visits with and without priapism by characteristics.

	**Priapism-related**	**Non-priapism-related**	**P-value**	
**Admission Rate**	49.80%	39.40%	0.001	
	**Priapism-related**	**P-value**	**Non-priapism-related**	**P-value**
**Region**				
Northeast	55.30%	0.37	44.50%	0.06
Midwest	49.40%		40.50%	
South	49.20%		37.60%	
West	39.60%		37.80%	
**Weekend**				
Yes	47.70%	0.16	38.20%	0.01
No	50.80%		39.90%	
**Season**				
Winter	55.40%	0.01	40.30%	0.02
Spring	53.40%		39.40%	
Summer	46.10%		38.70%	
Fall	45.30%		39.40%	

Additionally, SCD ED visits occurring on the weekend were less likely to result in hospital admission than those that occurred on a weekday (Table 2).

The percentage of admission from ED visits associated with priapism differed by season (Table 2). Among priapism-associated ED visits the percentage of admission was highest in the winter and spring months (55% and 53% respectively), and lower in the summer and autumn (46% and 45%).

### Top Complications and Co-occurring Conditions in Hospital Admissions

**Fig 1:**
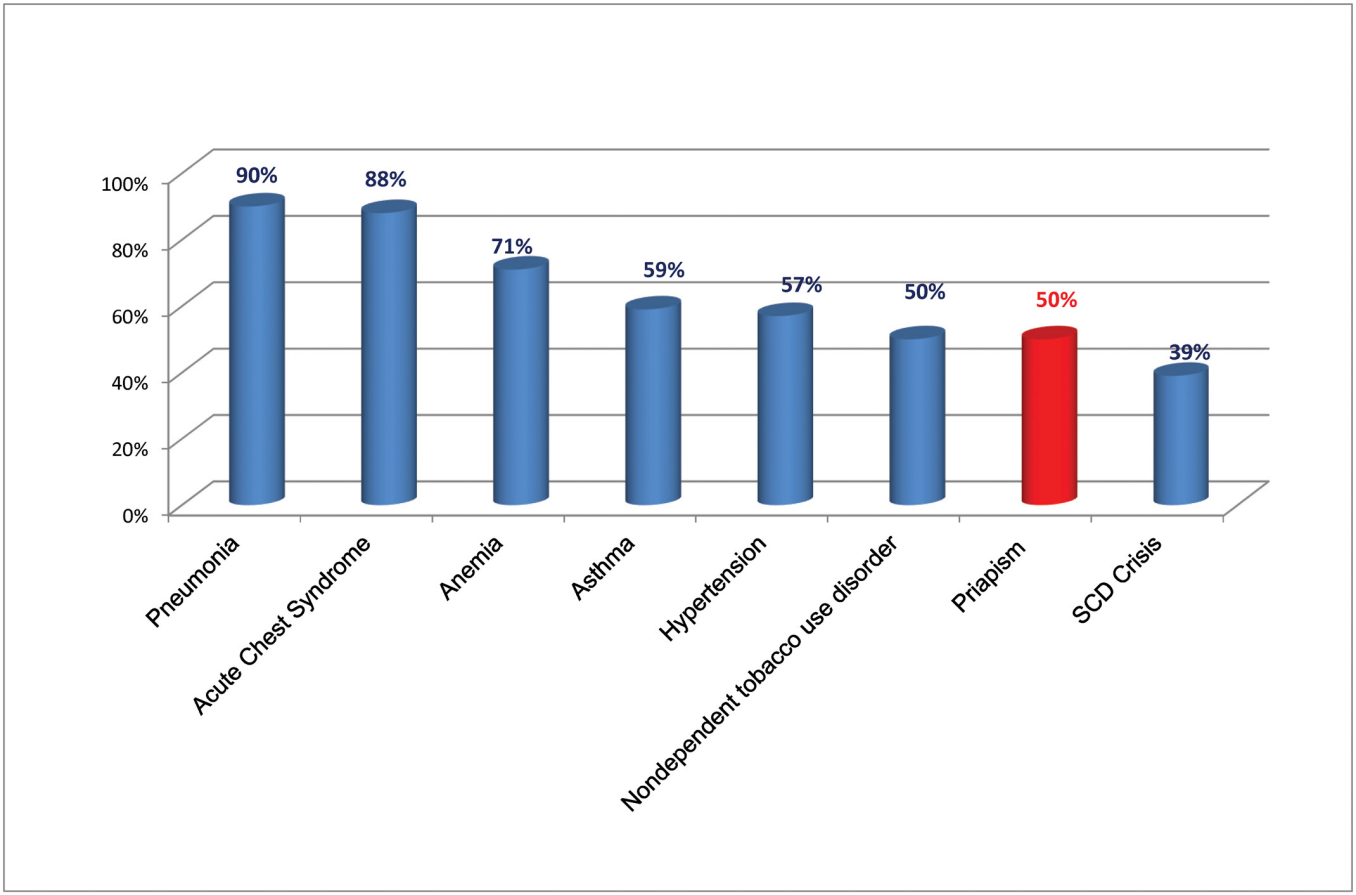
Percentage ED visits resulting in hospitalizations by top co-occuring conditions.

The overall percentage of hospital admission from ED visits among patients with SCD and priapism (50%) was relatively low compared with that among patients with SCD and relatively serious complications such as pneumonia (90%), anemia (71%), and acute chest syndrome (88%) (Fig 1).

Similar conditions were present in males regardless of priapism diagnosis **(**Table 3). Asthma and hypoxemia are the only co-occurring conditions that were considerably more common among admissions with priapism than other admissions from the ED with SCD. Asthma was present in 15% of admissions with priapism compared with 9% for other SCD-related admissions.

**Table 3:** The 10 most frequently co-occurring conditions based on *International Classification of Diseases*, *9*^*th*^ *Revision*, *Clinical Modification* codes resulting in hospital admission from the ED among patients with sickle cell disease by priapism status.

Priapism-Associated Admissions	Nonpriapism-Associated Admissions
Asthma (15%)	Pneumonia (12%)
Nondependent tobacco use disorder (13%)	Nondependent tobacco use disorder (12%)
Hypertension (10%)	Hypertension (10%)
Acute Chest Syndrome (7%)	Asthma (9%)
Pneumonia (7%)	Leukocytosis (8%)
Leukocytosis (7%)	Dehydration (7%)
Anemia (6%)	Acute Chest Syndrome (7%)
Dehydration (5%)	Anemia (6%)
**Hypoxemia** (5%)	Constipation (5%)
Constipation (5%)	**Hypokalemic** (5%)

## Discussion

One-half (50%) of ED visits for males with SCD and priapism resulted in inpatient admission, consistent with a previous report [15]. ED visits for males with SCD complicated by priapism were more likely to lead to a hospital admission than ED visits for a SCD crisis alone, although less than for severe acute SCD-related complications such as pneumonia, acute chest syndrome, and asthma.

The seasonality of hospital admissions with priapism and SCD is complex. On the one hand, ED visits with SCD and priapism were most frequent in summer months and least frequent in winter months, consistent with previous reports [14, 19]. On the other hand, the percentage of inpatient admission from an ED visit associated with priapism was lowest in summer and autumn and highest in the winter months. Consequently, the seasonal distribution of hospital admissions with priapism and SCD was even because of a lower rate of admission from the ED during the summer months for visits associated with priapism.

More research is needed to understand potentially modifiable factors that influence the rate of inpatient admission from the ED among persons with SCD and priapism.

The differential presence of diagnoses of asthma and hypoxemia with ED visits among males with SCD apparently provoked by priapism bears further investigation. Difficulties in breathing and low blood oxygenation could contribute to the occurrence of priapism. That finding suggests that targeting males with SCD who have asthma or other breathing problems for extra attention could potentially reduce the occurrence of episodes of priapism requiring acute care.

This study had several limitations. The observed frequencies of ED visits and admissions associated with SCD and priapism were based on ICD-9-CM codes that could have been misclassified or coded incorrectly. Previous studies have found coding errors in administrative data to be more common in non-inpatient records. For example, a recent study of Michigan children with SCD identified in newborn screening program records and linked to Medicaid claims data found that the percentage of false-positive ICD-9 codes for SCD in an ED claim was four times greater than in an inpatient claim, 16.5% vs. 3.9% [20].

Additionally, our analyses necessarily excluded priapism events that were resolved via home management, primary care, or spontaneously because only hospital-treated episodes were recorded. It is likely that many males with SCD suffer through stuttering episodes at home and do not present to the ED unless the event becomes intolerable. Consequently, the frequency of priapism among ED visits and inpatient admissions associated with SCD may primarily reflect the occurrence of major priapism events and not capture large numbers of stuttering priapism events managed at home or in an ambulatory setting.

The NEDS database does not allow patient identification. One implication is that there was no way to account for individual patients making multiple ED visits in a year. It is well known that a relatively small number of persons with SCD who are frequent visitors to the ED account for a large proportion of all SCD related ED visits. For example, in California, 40% of SCD-related ED visits in a single year were accounted for by 5% of patients [21]. Therefore, it is not possible to extrapolate from the share of ED visits among males with SCD with a priapism code to the percentage of individual patients with SCD who have one or more ED visits in a year had a visit associated with priapism. Another limitation of the NEDS data is that it is not possible to track patients to identify people at risk of developing complications, the calculation of incidence of complications, or to assess risk factors for complications.

Finally, in cases for which NEDS listed multiple ICD-9-CM diagnosis codes, it was impossible to determine which condition actually caused the ED visit or subsequent hospitalization. Because priapism is a profoundly acute and disabling event, our presumption is that ED visits in which priapism is coded were provoked by priapism.

### Conclusions

In this study, priapism was a complication observed relatively infrequently among males with SCD that visited the ED. However, ED visits for males with SCD that also had a priapism diagnoses were significantly more likely to result in hospitalization than ED visits for SCD related pain. This was especially true among males younger than 30 years of age. Although relatively few patients with SCD related ED visits had a priapism diagnosis in the ED, 35–40% of patients with SCD report having experienced priapism. It is likely that many of these males suffer through their stuttering episodes at home and do not present to the ED unless the event becomes intolerable. As a result, the ED visits that present in our study are likely the most severe, prolonged ischemic cases warranting shunt surgeries associated with hospital admissions.

Several factors that might warrant further research have been identified. Our ED observations are likely just a selective view of the entire problem, a better understanding of the patients that suffer from priapism that do not present to the ED is necessary. Additionally, it important to better understand risk factors for priapism associated with influences of environmental factors (ambient temperature, area of residence) or access to care (socioeconomic status, insurance type, area of residence).

## Disclaimer

The findings and conclusions in this report are those of the authors and do not necessarily represent the official position of the Centers for Disease Control and Prevention.
