# Correction: Emergency Department Visits and Inpatient Admissions Associated with Priapism among Males with Sickle Cell Disease in the United States, 2006–2010

**DOI:** 10.1371/journal.pone.0162056

**Published:** 2016-08-24

**Authors:** Brandi M Dupervil, Scott D Grosse, Arthur L Burnett, Christopher S Parker

The author middle initials were omitted from the published byline. The complete names are: Brandi M Dupervil, Scott D Grosse, Arthur L Burnett, Christopher S Parker.

The correct citation is: Dupervil BM, Grosse SD, Burnett AL, Parker CS (2016) Emergency Department Visits and Inpatient Admissions Associated with Priapism among Males with Sickle Cell Disease in the United States, 2006–2010. PLoS ONE 11(4): e0153257. doi:10.1371/journal.pone.0153257
